# Advanced glycation endproducts trigger autophagy in cadiomyocyte Via RAGE/PI3K/AKT/mTOR pathway

**DOI:** 10.1186/1475-2840-13-78

**Published:** 2014-04-14

**Authors:** Xuwei Hou, Zhaohui Hu, Hanying Xu, Jian Xu, Shunrong Zhang, Yigang Zhong, Xiuying He, Ningfu Wang

**Affiliations:** 1 Department of Cardiology, Hangzhou Hospital, Nanjing Medical University & Hangzhou First Municipal Hospital, Hangzhou 310006, China

**Keywords:** Cardiomyocyte, Autophagy, Advanced glycation end products, Hear function, Signal pathway

## Abstract

**Methods:**

Rat neonate cardiomyocytes were cultured and treated with AGEs at different concentration. Two classic autophagy markers, microtubule-associated protein 1 light chain 3 (LC3) and Beclin-1, were detected by western blot assay. The inhibition of RAGE and phosphatidylinositol 3-phosphate kinase (PI3K)/Akt/mTOR pathway were applied to cells, respectively.

**Results:**

AGEs administration enhanced the expression of Beclin-1 and LC3 II in cardiomyocytes, increased the number of autophagic vacuoles and impaired the cell viability in dose-dependant manners. Also, AGEs inhibited the PI3K/Akt/mTOR pathway via RAGE. Inhibition of RAGE with RAGE antibody reduced expression of Beclin-1 and LC3 II/I and inhibited the cellular autophagy, accompanied by the reactivation of PI3K/Akt/mTOR pathway in cultured cells. Notably, the presence of inhibition of PI3K/Akt/mTOR pathway abolished the protective effect of RAGE inhibition on cardiomyocytes.

**Conclusion:**

This study provides evidence that AGEs induces cardiomyocyte autophagy by, at least in part, inhibiting the PI3K/Akt/mTOR pathway via RAGE.

Previous studies showed that the accumulation of advanced glycation end products (AGEs) induce cardiomyocyte apoptoisis, leading to heart dysfunction. However, the effect of AGEs on another cell death pathway, autophagy, in cardiomyocytes remains unknown.

## Introduction

Advanced glycation endproducts (AGEs) are a group of heterogeneous compounds accumulated in diabetes due to factors including increased reactive carbohydrate substrate availability, oxidative condition favoring glycation and impaired detoxification [1]. AGEs form and accumulate in aging, renal failure, inflammation and especially diabetes mellitus (DM) [2]. Different cell membrane proteins have been shown to bind AGE and the best characterized receptor for AGE has been named receptors for advanced glycation end products (RAGE) [3]. The interaction between AGEs and RAGE participates in a variety of physiopathological process, including inflammation, carcinogenesis, atherosclerosis, nephropathy and neurodegeneration.

In diabetes mellitus, AGE/RAGE interaction contributes to the development of diabetic complications, including diabetic cardiomyopathy [4]. As one of the major complications of DM, diabetic cardiomyopathy is manifested by progressive heart failure and a poor prognosis in DM patients [5,6]. Previous studies had documented that the AGEs accumulation induces cardiomocyte apoptosis, leading to caricardiomocyte loss, is one of major mechanism leading to the development of heart dysfunction in diabetic cardiomyopathy [7].

Apoptosis is the type I programmed cell death pathway. Besides apoptosis, it also exists another type II pathway in mammal cells, namely, autophagy [8]. As a regulator of programmed cell death in mammals, autophagy is triggered by a variety of physiopathological stimuli, e.g. starvation, hypoxia, intracellular stress, hormones, ischemia and metabolism disorders [9]. Autophagy plays an essential role for mammal cell growth, survival, differentiation and development [10]. However, excessive autophagy becomes detrimental to cell fate, causing massive cell death and eventually leading to the function impairment *in vivo*[11-13].

Accumulating evidence indicate that autophagy is involved in the development of cardiovascular diseases. Autophagy is upregulated in almost all cardiac pathological states, exerting both protective and detrimental functions dependant on the extent of autogphagy [14]. Recent studies revealed massive presence of autophagic death in dead and dying cardiomyocytes in the failing hearts, including dilated cardiomyopathy, valvular heart diasese, hypertensive heart disease and chronic ischemia [15-18]. Notably, the presence of autophagic cardiomyocyte death in failing heart was more prevalent than that of apoptotic cells, suggesting an important role of autophagy in the cardiomocyte loss and heart function deterioration [19]. Excessive cardiac autophagy has been proposed as a maladaptive response that contributes to heart failure progression [20]. Autophagy may transform compensatory cardiac hypertrophy to pump failure. Diminished autophagy is reported to limits cardiac dysfunction in type 1 diabetes [21].

The phosphatidylinositol 3-phosphate kinase (PI3K) /Akt/mTOR signaling pathway is a well-known pathway involved in the regulation of autophagy in mammal cells [22]. A recent study showed that AGEs can inactivate Akt in rat vascular smooth muscle cells [23]. However, the effect of AGEs on cardiomocyte autophagy remains unknown. In this study, we sought to explore: 1. the role of AGEs in inducing cardiomocyte autophagy; 2. the possible signal pathway involved in the effect of AGEs on cardiomyocyte autophagy.

## Methods

### AGE preparation

AGE-bovine serum albumin (AGE-BSA) were prepared by incubating BSA (Sigma, St. Louis, MO) with 500 mmol/L of d-glucose under aerobic conditions for 10 weeks at 37°C in the presence of protease inhibitors and antibiotics based on published methods [24].

### Rat neonatal cardiomyocyte culture

For isolation and culture of Wistar rat neonatal cardiomyocytes, whole hearts from neonate rats (age less than 3 days) were isolated, minced and rinsed in hood. Five to six cycles of digestion using collagenase were performed. At the end of each cycle, the suspension was centrifuged and the supernatant collected, pooled, centrifuged and resuspended in the cardiac medium containing DMEM and M199 (volume ratio: 4:1). The medium was replaced every 48 h until cardiomyocytes reached >80% confluence for use.

### Cell treatment

Neonatal cardiomyocyte were treated with AGEs (10 mg/L, 25 mg/L, 50 mg/L and 100 mg/L for 48 hours). Also, anti-RAGE antibody (10 μg/ml, Abcam, USA) was used to block the binding of AGEs to RAGE. An mTOR inhibitor, Rapamycin (100 nM, Invitrogen, USA) was added to cultured cells to block the PI3K/Akt/mTOR in this study.

### Western blot assay

Cells were collected and lysed. The protein contents were determined. Samples were resolved by SDS-PAGE and electroblotted onto a polyvinylidene difluoride membrane. Afterward, membranes were incubated respectively with an anti-Belcin1(1:1000 dilution, Abcam, USA), anti-LC3(1:1000 dilution, Abcam, USA), anti-phosphorylated PI3K (p-PI3K, 1:1000 dilution, Abcam, USA) and total PI3K (t-PI3K, 1:1000 dilution, Abcam, USA), anti-phosphorylated Akt (p-Akt, 1:1000 dilution, Millipore, USA) and total Akt (t-Akt, 1:1000 dilution, Millipore, USA), anti-mTOR (1:1000 dilution, Millipore, USA) and anti-GAPDH antibodies 1:1000 dilution, Millipore, USA) overnight at 4°C, followed by incubation with the horseradish peroxidase-conjugated anti-rabbit IgG for 1 h at room temperature. The signals were enhanced using a chemiluminescence system (Amersham Pharmacia Biotech, Buckinghamshire, England).

### Cell viability MTT assay

Cardiomyocytes were plated in microtitre plate at a density of 3 × 10^5^ cells/mL. MTT was added to each well with a final concentration of 0.5 mg/mL, and the plates were incubated for another 2 h at 37°C and were quantified spectroscopically at 560 nm using a SpectraMax® 190 spectrophotometer.

### GFP-LC3 expression analysis and autophagosome quantitative analysis

For autophagy detection, 1×10^3^ cardiomyocytes were seeded into 96-well plates. Cultured cardiomyocytes were transduced with the adenovirus GFP-LC3 (Invitrogen, USA) at a multiplicity of infection of 60 for 24 h. The number of GFP-LC3 dots was determined by manual counting in 5 fields, and nuclear number was evaluated by counting DAPI-stained nuclei in the same fields using the same magnification. The number of GFP-LC3 puncta/cell was evaluated as the total number of dots divided by the number of nuclei in each microscopic field.

### Electron microscopy

Cardiomyocytes were fixed in 3% paraformaldehyde, 2.5% glutaraldehyde, and 0.1 m cacodylate buffer (pH 7.4). After washing with the buffer solution and post-fixation in 1% OsO4 and 0.1 m cacodylate buffer (pH 7.4), they were washed with the buffer solution, dehydrated using alcohol and acetone, and embedded in epoxy resin. Ultrathin sections were examined under the electron microscope.

### Statistic analyses

Data are presented as mean ± S.E.M. Data were analyzed using a one-way ANOVA followed by the S-N-Keuls post-hoc analysis. To evaluate the amount of protein expression, the Raytest TINA software (http://www.raytest.de/service/raytest_catalog.html) was used for western blotting to calculate the densitometric analysis. The differences in the relative expressions among different genotypes were compared by using ANOVA analyses. The analyses were performed with the SPSS software (Statistical Package for the Social Sciences, version 16.0, SPSS Inc, Chicago, IL, USA).

## Results

### AGEs increase RAGE, Belcin1 and LC3-II expressions

**Figure 1:**
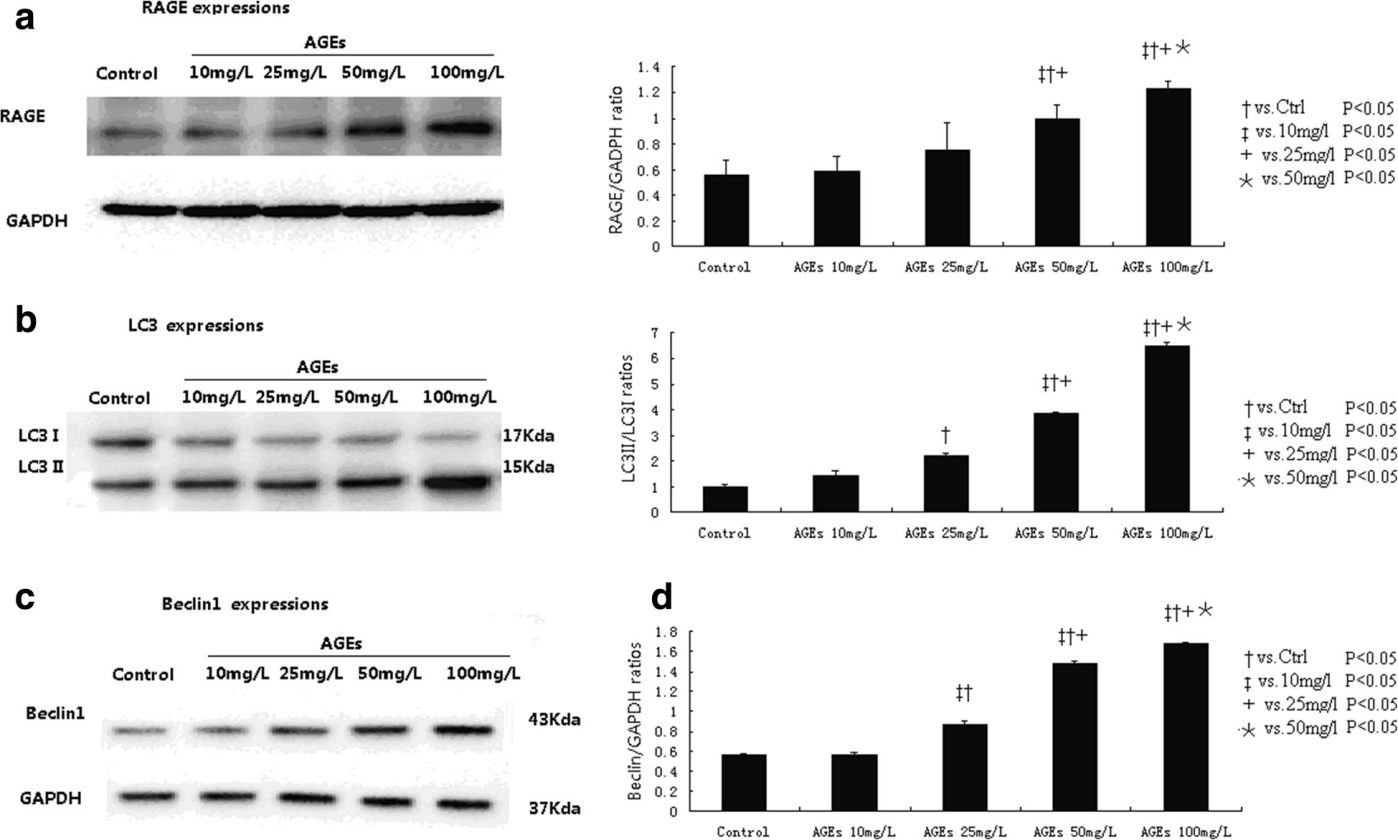
**AGEs affect the expressions of RAGE, Belcin1 and LC3-I and LC3-II expressions in cultured cardiomyocytes.** AGEs administration stimulated the expressions of RAGE **(a)**, LC3 **(b)** and Beclin 1 **(c)** in dose-dependant manners.

Figure 1 shows the RAGE, LC3 and Beclin-1 protein expressions after AGEs treatment at different concentrations. LC3 shows two bands on western blot assay, namely, LC3-II and LC3-I (Figure 1a). The relative expression ratio between LC3-II and LC3-I (LC3-II/LC3-I) is used to evaluate the extent of autophagy. Our results showed that the AGEs administration stimulated the expressions of RAGE, Beclin 1 and LC3 II in dose-dependant manners. Quantitative analyses revealed that the relative expression ratios of RAGE/GAPDH, LC3-II/LC3-I and Beclin1/GAPDH increased in cardiomyoctye with AGEs concentrations (Figure 1a-1c).

### AGEs treatment induces typical autophagic changes

**Figure 2:**
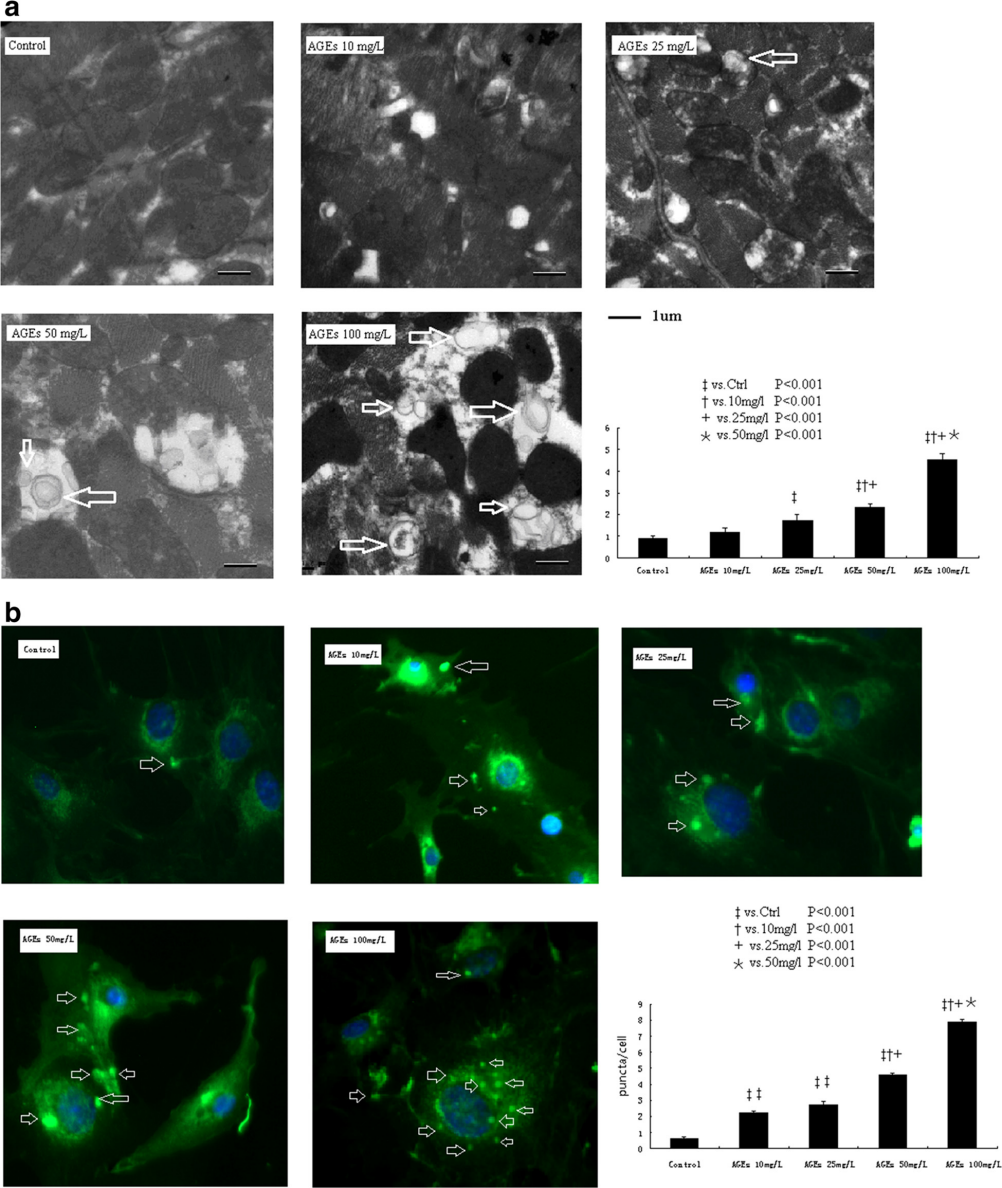
The representative figures of double-membrane vacuoles under electron microscopy observation (arrow heads, 2a) and intra-cellular GFP-LC3 redistribution (green spots, 2b) in cultured cardiomyocytes treated with different AGEs concentration.

Double-membrane vacuoles are the typical morphological changes in autophagy under electron microscopy observation. Figure 2a shows the typical double-membrane vacuoles in cultured cardiomyocytes. The presence of autophagic double-membrane vacuoles was rare in control cardiomyocytes (Figure 2a). With the increase of AGEs concentrations, the presence of autophagic vacuoles became progressively prevalent. For quantitative analyses, we counted the appearance of double-membrane vacuoles in 5 random fields for each sample. The mean number of autophagic vacuoles gradually increased with the concentration of AGEs (2.1 ± 0.9 at 10 mg/L; 2.7 ± 0.1 at 25 mg/L; 3.3 ± 0.4 at 50 mg/L;4.5 ± 0.3 at 100 mg/L; P < 0.001).

The formation of autolysosome in cultured cardiomyocytes was detected by intra-cellular GFP-LC3 redistribution and calculated as the average number of LC3-labeled puncta per cell (Figure 2b). Compared with controls, AGEs augmented the average number of LC3-labeled puncta per cell with the increase of its concentrations (2.2 ± 0.13 at 10 mg/L; 2.7 ± 0.20 at 25 mg/L; 4.6 ± 0.13 at 50 mg/L and 7.9 ± 0.19 at 100 mg/L, P < 0.001). Collectively, these results suggest that AGEs induced autophagy in cultured cardiomyocyte in a dose dependant manner.

### AGEs impair cardiomyocyte viability

**Figure 3:**
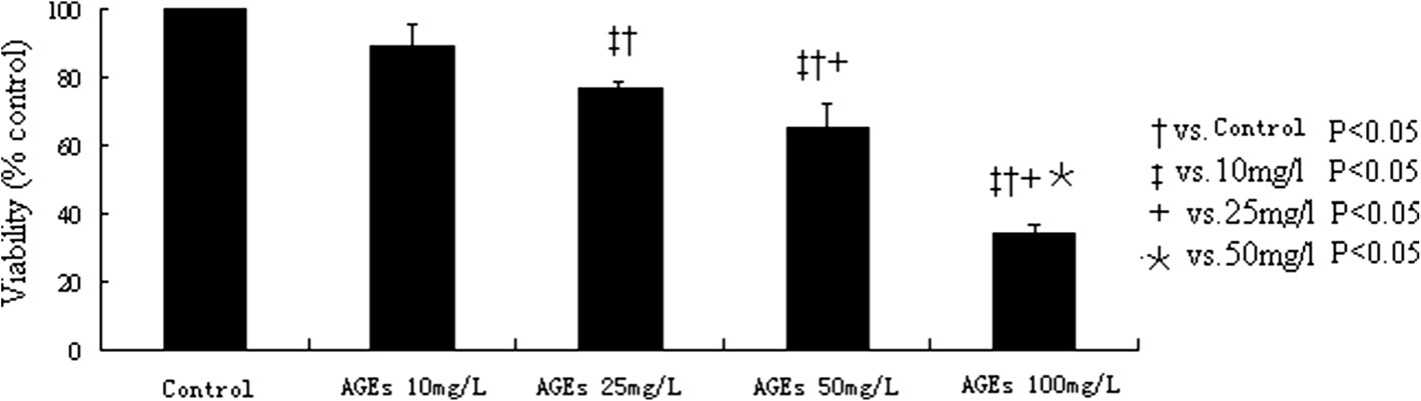
**The effects of AGEs exposure on the cardiomyocyte viability.** MTT assay shows that AGEs impair the cardiomyocyte viability in a dose-dependant manner.

We next tested the effects of AGEs exposure on the cardiomyocyte viability. MTT assay shows that AGEs impair cardiomyocyte viability in a dose-dependant manner (Figure 3). With the increase of AGEs concentration, the cell viability decreased progressively and reached a lowest level at AGEs 100 mg/L.

### AGEs inhibits the PI3K/Akt/mTOR pathway via RAGE

**Figure 4:**
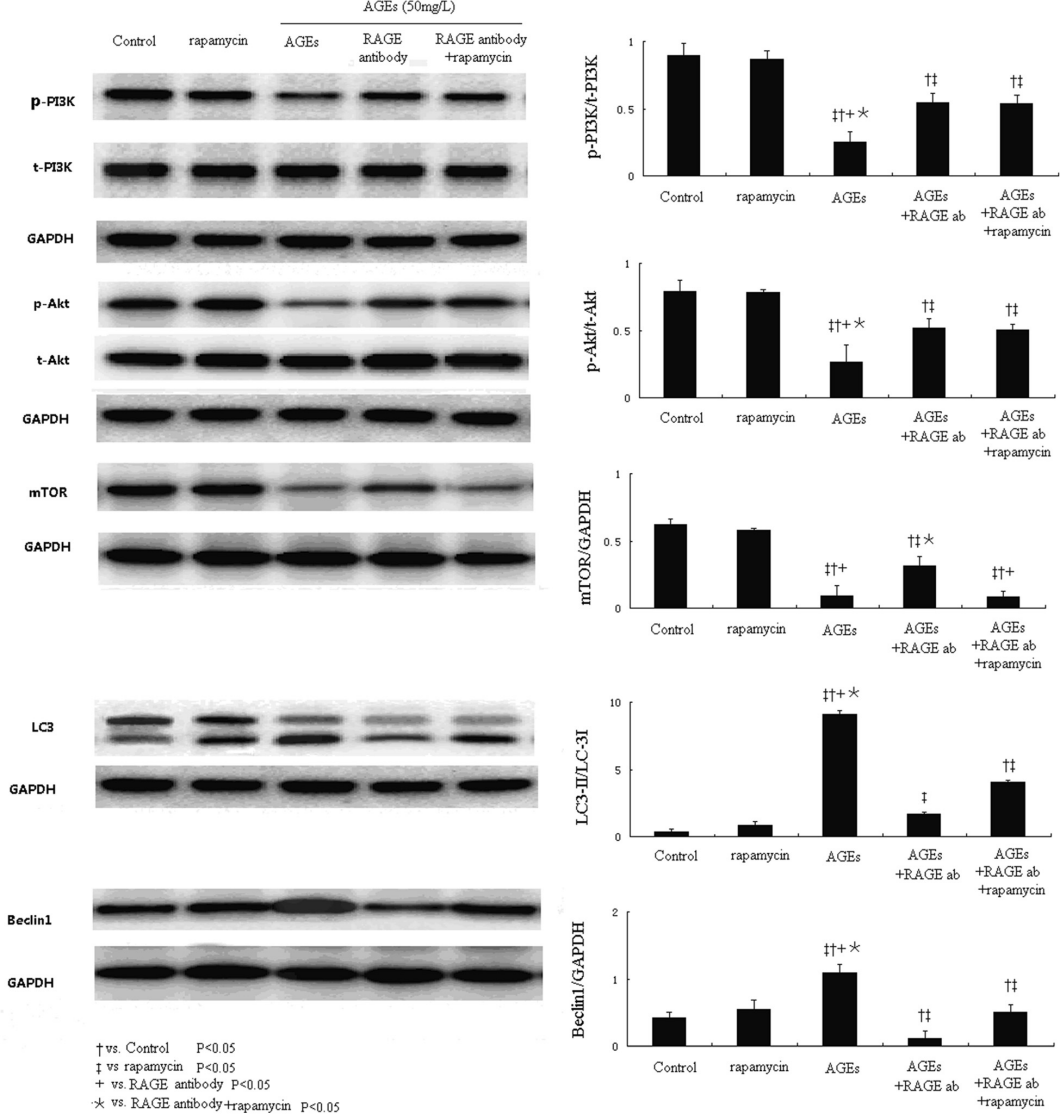
**The effect of AGEs treatment on RAGE ,PI3K/Akt/mTOR pathway in cultured cardiomyocytes.** AGEs treatment stimulated the RAGE expression, however, inhibited the PI3K/Akt/mTOR pathway in cultured cardiomyocytes compared to controls. RAGE antibody pretreatment re-activated the PI3K/Akt/mTOR pathway and reduced Beclin1 and LC3-II. Interestingly, the addition mTOR inhibitor rapamycin increased the Beclin1 and LC3 expression again compared to cell receiving RAGE antibody pretreatment.

Western blot assay shows that the AGEs treatment stimulated the RAGE expression, however, inhibited the PI3K/Akt/mTOR pathway in cultured cardiomyocytes compared to controls (Figure 4). When RAGE was blocked by RAGE antibody pretreatment, the PI3K/Akt/mTOR pathway was re-activated indicated by the increased expression ratios of p-PI3K/t-PI3K, p-Akt/t-Akt and mTOR/GAPDH with RAGE blockage. These results suggest AGEs deactivate the PI3K/Akt/mTOR pathway via its receptor RAGE.

### Inhibition of AGEs and PI3K/Akt/mTOR pathway on autophagy level

We next test the role of PI3K/Akt/mTOR pathway in AGEs induced autophagy in cardiomyocytes by using mTOR inhibitor, rapamycin. Figure 4 show that rapamycin dramatically inhibited the mTOR expression and increased the Beclin1 and LC3-II expression levels, without affecting the expression of RAGE, PI3K and Akt. As mentioned above, RAGE antibody pretreatment activated the PI3K/Akt/mTOR pathway, and markedly brought down the autophagy marker Belcin1 and LC3-II expressions. However, when cells were co-treated with rapamycin, the mTOR expression induced by RAGE antibody was inhibited, accompanied by increased Belcin1 and LC3-II expressions.

**Figure 5:**
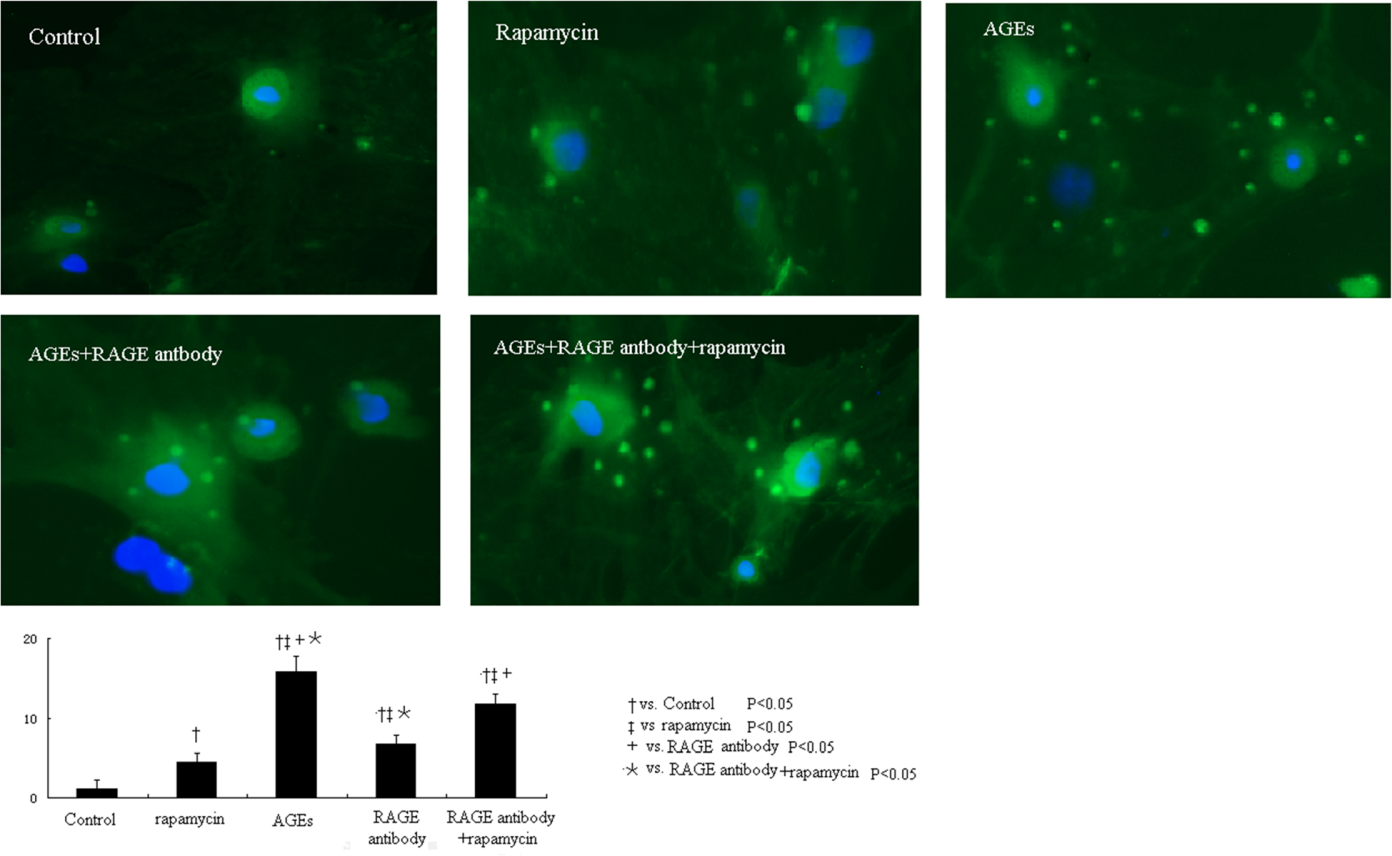
**Compared to control cells, compared to control cells, rapamycin along increased the autophagy level indicated by the number of average number of LC3-labeled puncta per cell (Figure **5**).** RAGE antibody pretreatment markedly reduced the formation of autolysosome in cultured cardiomyocytes, which can be induced again by the addition of rapamycin.

We performed the GFP-LC3 assay in cultured cells. Compared to control cells, rapamycin along increased the autophagy level indicated by the number of average number of LC3-labeled puncta per cell (Figure 5). AGEs at 50 mg/L significantly prompted the LC3 translocation, while the RAGE antibody pretreatment markedly reduced the formation of autolysosome in cultured cardiomyocytes. Interestingly, when these cells were treated with rapamycin, the number of LC3-labeled puncta per cell was increased again (Figure 5). These results provide direct evidence to support the involvement of RAGE/ PI3K/Akt/mTOR pathway in AGEs induced autophgy in cardiomyocytes.

### Inhibition of RAGE and PI3K/Akt/mTOR pathway on cell viability

**Figure 6:**
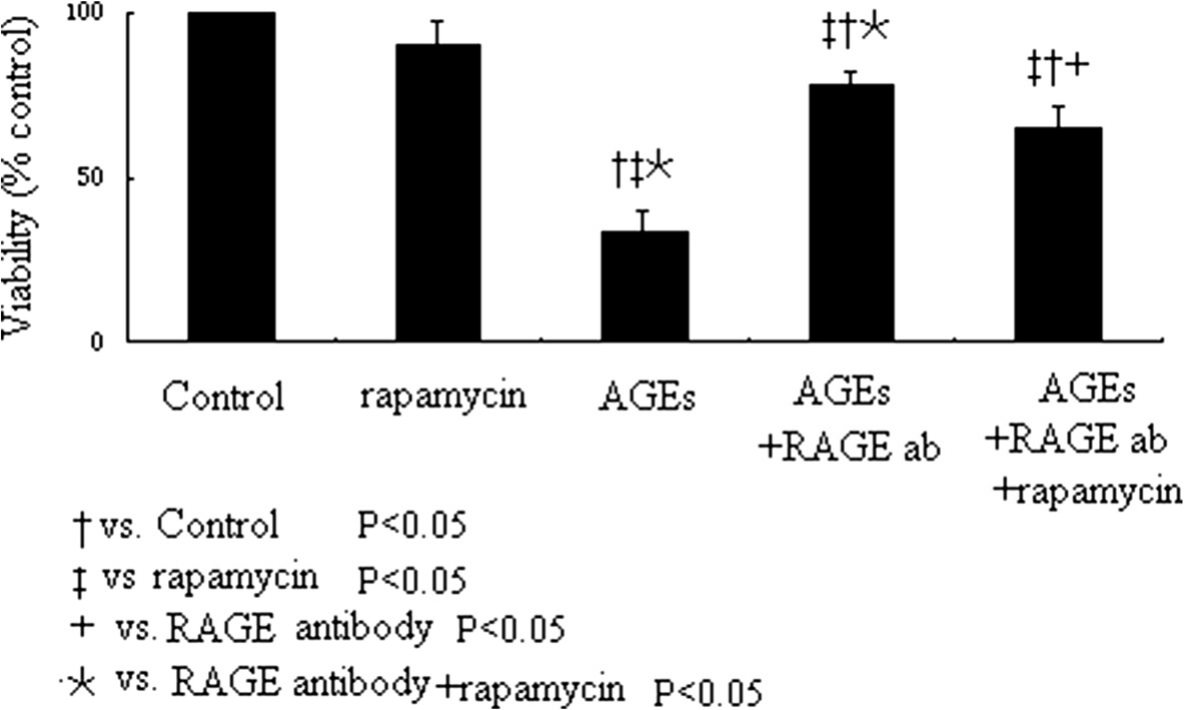
**Cardiomyocyte viability after the Inhibition of RAGE and PI3K/Akt/mTOR pathway on cell viability.** Compared to control cells, AGEs treatment dramatically reduced the cell viability, but rapamycin along did not significantly change the cell viability. Inhibition of RAGE by its antibody can rescue cell viability induced by AGEs treatment; however, this beneficial effect was abolished by additional rapamycin treatment.

MTT assay evaluated the cardiomyocyte viability after the Inhibition of RAGE and PI3K/Akt/mTOR pathway on Cell viability Figure 6. Compared to control cells, rapamycin along did not significanlty change the cell viability. RAGE antibody dramatically improved the cell viability induced by AGEs treatment; however, rapamycin addition to these cells will impair the cell viability.

## Discussion

In the present study, we found that AGEs administration enhanced the expression of Beclin-1 and LC3 in cardiomyocytes, increased the number of autophagic vacuoles and more importantly, impaired the cell viability in dose-dependant manners. Inhibition of RAGE with sRAGE pretreatment reduced the Beclin-1 and LC3 II expressions, ameliorated the cardiomyocyte ultra-structure and improved the cell viability to AGEs exposure. Furthermore, we found that AGEs inhibits the PI3K/Akt/mTOR pathway via RAGE, while blockage RAGE re-activated PI3K/Akt/mTOR pathway, however, the addition with PI3K inhibitor redeuced the cell autophagy and improve the cell viability. Our data suggest that the AGEs induced cardiomyoctye autophagy, at least in part, via RAGE/PI3K/Akt/mTOR pathway. As far as we know, this is the first study reporting the effect of AGEs on autophagy of cardiomyocytes.

Recently the heart function losses induced by excessive autophagy attract much more attention. Autophagy was reported to be detected in cardiomyocytes from patients with aortic stenosis and dilated cardiomyopathy [25-28]. Like in other tissues, autophagy in the heart under baseline conditions is a homeostatic mechanism for maintaining cardiomyocyte size, global cardiac structure and function.

Autophagy has been observed in both hypertrophied myocardium [29] and failing myocardium with a variety of etiology, including dilated cardiomyopathy [30,31] valvular disease [32], and ischemic heart disease [33,34]. In idiopathic dilated cardiomyopathy, the prevalence of autophagic cells are 40 times more than apoptotic cells [19], suggesting importance of autophagic death in heart function maintenance. Myocardial autophagy is reported to contribute to post-burn cardiac dysfunction in rat model and enhanced autophagy with Rapamycin decreased cardiac functon while inhibits autophagy with 3-MAprotects the cardiac function [35]. In this study, we found that the AGEs induced autogphagy and contribute to the impairment of cell viability. This finding suggests that the pharmacological regulation of autophagy may be an anther plausible strategy to prevent the progressive heart function loss in high AGEs setting, e.g. DM patients.

Another novelty of this study was that we investigated the signal transduction pathway under which AGEs induced autphagy in cardiomyocytes. The effect of AGEs on PI3K/Akt/mTOR pathway is quiet controversial. AGEs stimulated phosphorylation of Akt in macrophages [36] and triggers activation of the PI3K pathways in mesangial cells [37]. In contrast, AGEs upregulate the RAGE expression, but downregulate the Akt activity late endothelial progenitor cells [38]. In rat vascular smooth muscle cells, AGEs treatment inhibits the phosphorylation of Akt and mTOR [23]. In the present study, we showed that AGEs induced the autophagy in cardiomyoctyes via suppressing the classic PI3K/Akt/mTOR pathway. More importantly, this modulation of this pathway led to functional changes in cell viability, suggesting that this pathway may be the pharmacological target in the regulation of autophagy in cardiomyocytes.

Limitations should be addressed in current study. Firstly, we only studied the effect of AGEs perfusion on cardiomyocyte autophagy and did not assess its effect on cardiomyocyte apoptosis. We did not compare the contribution of cardiomycyte autophagy and apoptosis in heat function loss in this study. Secondly, it should be pointed out that the PI3K/Akt/ mTOR signal pathway is not the only regulator of also modulate the autophagy. We did not study the other signal pathway in this study.

## Competing interests

The authors declared that they have no competing interests.

## Authors’ contributions

XWH designed the study and carried out the in vitro cell studies and autophay detection.ZHH prepared the AGEs and cell culture and treatment.HYX, JX, SRZ,XYH and NFW were involved in the signal pathway detection. XWH performed the statistic analyses and wrote the manuscript. All authors read and approved the final manuscript.
